# Identification of cell-type-specific, transcriptionally active transposable elements using long-read RNA-sequencing data-based comprehensive annotation

**DOI:** 10.1186/s44342-025-00048-1

**Published:** 2025-08-06

**Authors:** Chaemin Lim, Hyunsu An, Jihwan Park

**Affiliations:** School of Life Sciences, Gwangju Institute of Science and Technology (GIST), Gwangju, 61005 Republic of Korea

**Keywords:** Single-cell RNA-sequencing, Long-read RNA-sequencing, Transposable element, Transcriptome annotation, De novo transcript

## Abstract

**Background:**

The biological functions of transposable element (TE)-derived transcripts during physiological development, disease development, and progression have been previously reported. However, research on locus-specific TE-derived transcript expression in various human cell types remains limited.

**Methods:**

We processed 2596 publicly available human long-read RNA-sequencing (LR RNA-seq) datasets covering 21 organs and 71 cell lines in both healthy individuals and diseased patients with various conditions to compile this TE-derived transcript annotation. We established a pipeline for assembling transcripts containing TE sequences to measure transcriptionally active TE-derived transcripts in diverse tissues and cell types. Next, we applied our TE annotation to the Genotype-Tissue Expression (GTEx) single-cell RNA-sequencing (scRNA-seq) data from eight tissues.

**Results:**

We constructed the first transcriptom6e-based TE annotation using massive amounts of human LR RNA-seq data for use as a comprehensive reference to detect locus-specific TE-derived transcripts. Our annotation showed better detection accuracy for TE-derived transcripts than the RepeatMasker and GENCODE nonTE gene annotations. This annotation enabled the identification of novel TE-derived transcripts and their isoforms. We also identified alternative transcription end sites for long noncoding genes and confirmed previously annotated TE-nonTE gene fusion transcripts. Next, we applied our TE-derived transcript annotation to public scRNA-seq data from various human tissues and identified several cell-type-specific TE-derived transcripts in a locus-specific manner.

**Conclusions:**

We generated a comprehensive, TE-derived transcript annotation using large-scale, LR RNA-seq data. Researchers can use our TE reference annotation to analyze active TE transcripts and their splicing isoforms in specific transcriptome datasets and to detect de novo TE transcripts. The discovery of cell-type-specific TE-derived transcripts may help explain mechanisms underlying the maintenance of cellular identity and provide new insights into the pathological mechanisms of various diseases.

**Supplementary Information:**

The online version contains supplementary material available at 10.1186/s44342-025-00048-1.

## Background

Once regarded as “junk DNA,” transposable elements (TEs) are now known to comprise nearly 50% of the human genome [1]. However, advancements in next-generation sequencing (NGS) technology have revealed their crucial roles in gene regulation, chromatin dynamics, and diverse biological processes across various physiological and disease states [2–6]. During mammalian development, specific TE families exhibit stage-specific expression patterns and regulate key developmental genes by interacting with partner proteins and transcription factors [7, 8]. Moreover, long terminal repeat (LTR) retrotransposon transcripts contribute to epigenetic changes by influencing the three-dimensional structure of chromatin during development [3]. Conversely, certain TE-derived transcripts may have deleterious effects. One notable example is onco-exaptation, in which a TE promoter aberrantly activates an oncogene, thus contributing to tumorigenesis [6, 9–11]. Given these findings, it is reasonable to hypothesize that TE-derived transcripts play a critical role in defining cellular identity and regulating the biological functions of each cell type.

As single-cell RNA-sequencing (scRNA-seq) has become an essential technique for exploring cellular behavior [12–14] and research on TE-derived transcripts has gained momentum, several studies have developed methods to accurately quantify and assess the effects of TE-derived transcripts at the cellular level [15–18]. The single-cell TE processing pipeline scTE was designed to analyze the TEs in scRNA-seq datasets and show the TE expression patterns across diverse cell types [15]. However, it groups TE expression by family level but does not take into account genomic location. SoloTE addresses this limitation by using mutation information to distinguish individual TEs within the same family. However, its locus-specific TE information is still based on predictions rather than experimentally observed expression, which is a key indicator of transcription activity [16]. CELLO-seq can detect active TE-derived transcripts at single loci using in-house long-read RNA-seq (LR RNA-seq)-based TE-derived transcript annotations [17]. However, the CELLO-seq annotation contains TE-derived transcripts that are expressed exclusively in mouse blastomeres. Consequently, a comprehensive system that is applicable to various cell types and incorporates information on transcriptionally active TE-derived transcripts in humans is required.

Long-read sequencing technologies are capable of sequencing DNA or RNA molecules ranging from several kilobases (kb) up to hundreds of kilobases and are widely used for applications such as de novo assembly, structural variation detection, repeat sequence mapping, and splicing variant analysis. Among these applications, transcriptome profiling using LR RNA-seq has emerged as a particularly powerful approach to overcome the limitations of measuring TE activity at the single-cell level [18–20]. LR RNA-seq produces complete transcript sequences, offering a notable advantage in identifying repeat sequences like TEs that are hard to detect with short-read RNA-sequencing due to their repetitive nature and uncertain genomic position [21–23]. Additionally, LR RNA-seq data offer certain advantages over UCSC Genome Browser RepeatMasker annotations, which are commonly used to quantify TE-derived transcripts using repeat sequence databases [24, 25]. Although RepeatMasker provides a comprehensive overview of TE-containing regions, it fails to capture the detailed structure and expression levels of TE-derived transcripts because it lacks transcript and exon information. This restriction was a result of the initial intention to mask duplicated sequences, specifically to enhance the accuracy of short-read mapping and nonTE gene analysis. As a result, RepeatMasker annotations may include “fossilized” TE-derived transcripts with no current transcriptional activity. This issue is particularly problematic when analyzing SR RNA-seq or scRNA-seq data, in which the high frequency of short reads increases the risk of false-positive mapping to inactive TEs.

In the present study, we assembled a TE-derived transcript annotation and explored the features of the TE-derived transcripts in our annotation. We applied this annotation to a broad and diverse scRNA-seq dataset encompassing various tissues and cell types. Through this analysis, we identified TE-derived transcripts specific to certain cell types that have potential as novel cell type markers.

## Methods

### Data collection and preprocessing

The 2596 SRA sequencing experiment entries that utilized the 2 most popular LR RNA-seq technologies, PacBio and Oxford Nanopore Technologies, were retrieved from the NCBI SRA database (https://www.ncbi.nlm.nih.gov/sra) using the search queries ‘human’, ‘long-read’, ‘rna-seq’, ‘nanopore’, and ‘pacbio’ [26]. The FASTQ files were downloaded from the NCBI SRA database using SRA Toolkits (v3.0.0). The FASTQ files were aligned against the GRCh38 genome using minimap2 (v2.24) with the “splice” preset and default settings [27].

### The establishment of TE selecting pipeline

The computational pipeline for enriching and classifying TE-overlapped reads for long- and short-read sequencing data was selected as follows. Initially, the individual reads from the input BAM file were processed using the Pysam (v0.19.0) AlignmentFile object [28]. Then, reads above the mapping quality threshold (30 was used in this study) were further analyzed in the pipeline. Hard-clipped reads were excluded because minimap2 (v2.24) typically realigns reads with poor mapping quality using soft-clipping, shortening the reads through hard clipping. Next, a binary mask of an entire human genome was generated with the bitarray (v2.5.1) package. Genomic positions that coincided with repeat elements (TEs) based on the UCSC Genome Browser RepeatMasker repeat annotation (GRCh38) were assigned a value of 1, while all remaining regions of the genome were assigned a value of 0. The binary masks were then used to efficiently select reads overlapped with valid TE regions. Similarly, a binary mask of all exons in the reference gene annotation was constructed, which was used to further classify TE-overlapped reads. The TE-overlapped reads were grouped into four categories, with one category designated as a catch-all category. The first category is an intergenic TE-containing transcript, capturing reads that did not overlap with a previously annotated nonTE gene but only overlapped with an intergenic TE region. The second category is TE-containing transcript overlapping with a nonTE gene, which captures reads that were overlapped with a nonTE gene but also shows a significant overlap with an intragenic TE region (the overlapped base pairs should be > 80% of the total aligned length). The third category is TE-nonTE fusion transcript that captures reads overlapping with a nonTE gene, as well as an intergenic TE region. The last category, as previously described, collects all reads that were not classified into the aforementioned three categories.

### Transcriptome assembly and annotation of TE-derived transcripts

Filtered TE-derived reads were assembled using Stringtie2 (v.2.1.7) [29]. We ran Stringtie2 with the following parameters (-L -G -v -p 12 -j 1 -g 0 -f 0.01 -l STRG -m 50 -t -c 1.5 -s 1.5). To construct a reference TE-transcript annotation for guiding TE transcriptome assembly, we utilized the GENCODE reference nonTE gene annotation from the Ensembl database (release version 105, downloaded from asia.ensembl.org/info/data/ftp/) and the UCSC Genome Browser RepeatMasker repeat annotation (GRCh38) [24, 25, 30]. After completing the assembly of TE-derived reads into TE-derived transcripts, each TE-derived transcript was annotated by combining a unique label generated by Stringtie2, such as STRG1, with a list of TE subfamily names that overlapped with the TE-derived transcript. When multiple TE subfamily sequences overlapped with a single transcript, we sorted the TE subfamily names in decreasing order based on the percentage of the transcript that overlapped with the TE elements of the TE subfamily annotated in the RepeatMasker annotation.

### Validation of splicing event

Splicing acceptor and donor sequence motif analysis was performed using the Python package Logomaker [31]. Three annotations were used as inputs: GENCODE reference nonTE gene annotation, TE-derived transcript annotation, and TE-derived transcript annotation without transcripts overlapping nonTE genes [24, 30]. First, we constructed a count matrix from sequence alignment, which contains sequence information matching each target locus of the transcript in each annotation file using Logomaker.alignment_to_matrix function. In this analysis, the splicing acceptor sites and splicing donor sites are the target loci. Next, each constructed count matrix is transformed into an information content matrix to display the frequency of nucleotides at each position using the Logomaker.transform_matrix function. Finally, the information content matrix was visualized using Logomaker.Logo function.

### scRNA-seq data analysis

#### Processing the GTEx single-nucleus reference database

The GTEx project (dbGaP accession phs000424) provides raw and processed data on the AnVIL workspace “AnVIL_GTEx_V8_hg38” [32]. The 10× CellRanger output files for the GTEx single-nucleus reference database were downloaded from the Google Cloud Platform (GCP) using the gsutil tool (v5.5). In total, 102 BAM files (955 GB) were downloaded.

#### Quantification of the isoforms of TE-derived transcripts in scRNA-seq data

To quantify the isoforms of TE-derived transcripts at single-cell resolution, TE-derived transcripts that overlapped with non-TE genes in the latest GENCODE reference were filtered out as a conservative step [30]. This approach ensured that reads mapping to regions shared by TE-derived and nonTE gene annotations were assigned to the nonTE gene annotations, effectively excluding them from the analysis. The refined TE-derived transcript annotations were then integrated with the reference nonTE gene annotations, creating a comprehensive set for quantifying gene and isoform expression at the single-cell level.

The barcoded BAM files (one of the output files from the 10× CellRanger program) were downloaded from the GTEx project. We scanned the provided barcoded BAM file using the Pysam module (v0.19.0) and assigned valid reads to genes, isoforms, and RepeatMasker elements using the interval tree module (v3.1.0) [28]. The quantification results are then exported as a count matrix, resulting in a collection of files that represent a 10×-formatted feature-barcode count matrix.

#### Preprocessing of the single-cell expression data

The 10×-formatted output count matrix was read into a Python (v3.8) environment using the Scanpy (v.1.9.1) package [33]. The cell annotations were imported from the processed scRNA-seq data (“GTEx_8_tissues_snRNAseq_immune_atlas_071421.public_obs.h5ad”) downloaded from the GTEx portal (https://gtexportal.org/). The matching cell barcodes were identified through the assignment of 10× channels available in the metadata of the processed single-cell data to the barcoded BAM files analyzed in the current study. Cell barcodes not matching the metadata were discarded. Quality control and basic downstream analyses were performed as in our previous studies [34, 35]. The resulting AnnData was log-normalized with the default settings using the sc.pp.normalize_total function and sc.pp.log1p function, and expression values of the identified highly variable features were determined using the default settings using sc.pp.highly_variable_genes function. These highly variable genes were used to reduce dimensions using principal component analysis (PCA) implemented by the sc.tl.pca function and to create a Uniform Manifold Approximation and Projection (UMAP) embedding of the 174,419 cells using the sc.tl.umap function.

#### Identification of cell type-specific TE-derived transcripts and isoforms

Cell type-specific genes, including nonTE genes, isoforms of nonTE genes, TE-derived transcripts, and isoforms of TE-derived transcripts, were identified by the Wilcoxon rank-sum test using the scanpy.tl.rank_genes_groups function. Cell type-specific TE-derived transcripts and TE-derived isoforms were extracted based on the presence of a gene_id that includes “STRG.” Cell type-specific TE-derived transcripts were identified with adjusted *p*-values < 0.05. Cell type-specific TE-derived transcripts were visualized in a UMAP-based scatter plot using the scanpy.pl.umap function.

## Results

### Constructing transcript annotation derived from TE using LR RNA-seq data

**Fig. 1:**
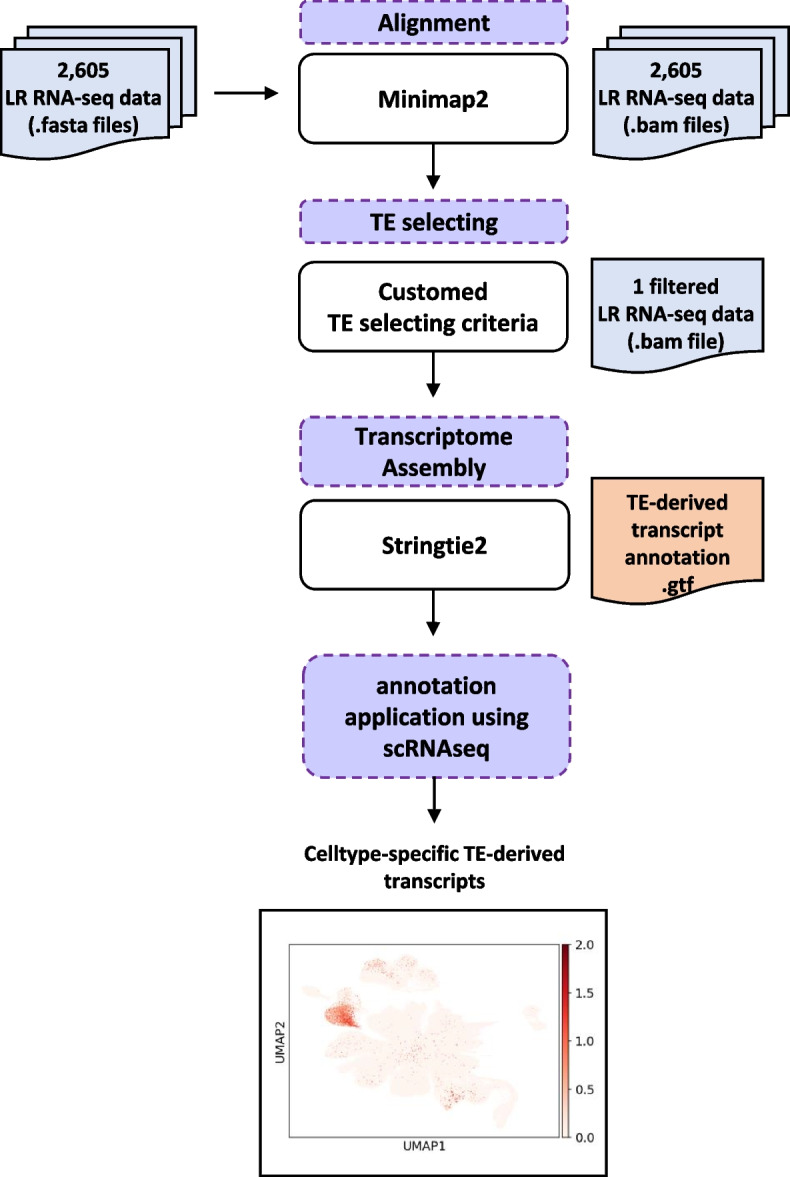
Workflow for the construction of the transposable element (TE)-derived transcript annotation. The workflow demonstrates the stepwise approach to constructing a TE-derived transcript annotation. Starting with 2596 publicly available long-read RNA-sequencing (LR RNA-seq) data in FASTA format, alignment is performed to process raw sequencing reads. Next, TE selection is applied to filter and identify TE-derived transcripts, resulting in a single-filtered BAM file. Transcriptome assembly is conducted to generate the final TE-derived transcript annotation in GTF format. Finally, the annotation is applied to single-cell RNA-sequencing (scRNA-seq) data for further downstream analysis, as illustrated in the Uniform Manifold Approximation and Projection (UMAP) visualization at the bottom

To construct a TE-derived transcript annotation, we collected publicly available LR RNA-seq data from the Sequence Read Archive (SRA) (Fig. 1) [26]. A total of 2596 LR RNA-seq raw datasets were downloaded, and the sequencing reads were mapped to the human reference genome using Minimap2 [27]. Then, we selected all LR reads overlapping with TE sequences but not with nonTE repeat sequences, such as transfer RNA sequences (tRNAs) and simple repeats, as identified by RepeatMasker. Next, the reads were classified into four categories based on their genomic location relative to nearby coding genes and RepeatMasker elements: (1) intergenic, (2) intragenic, (3) spanning both TE and nonTE genes, and (4) all other reads overlapping TE sequences (Supplementary Fig. 1). The first category, intergenic TE-containing transcripts, included reads that overlapped only with an intergenic TE region and did not overlap with any nonTE genes. The second category, intragenic TE-containing transcripts, consists of reads that overlap with both a nonTE gene and an intragenic TE region, with more than 80% of the reads aligned to the TE region. The third category included reads that overlapped with both a nonTE gene and an intragenic TE region, similar to the second category, but also overlapped with an intergenic TE region, suggesting the potential for a TE-nonTE gene fusion transcript. Finally, the fourth category included all the remaining reads that did not fit the previous three categories. We observed that TE-derived reads comprised 15.6% of the total uniquely mapped reads, and the majority of the TE-derived reads (12.3%) were not included in the first three categories in the entire set of 2596 LR RNA-seq samples (Supplementary Fig. 2a). To further explore whether the proportions of these four categories vary by tissue, we examined the distributions of tissues and found that although some variation was observed, the patterns were largely consistent across tissues (Supplementary Fig. 2b). We decided to exclude TE-derived reads produced from criterion 4 in order to construct a high-confidence TE-derived transcript annotation, as such reads ambiguously span both TE and non-TE gene regions, making their transcript origin uncertain. We then used Stringtie2 [29], a transcriptome assembler, to construct a TE-derived transcript annotation (Supplementary Data S1).

### Summary of the TE-derived transcript annotation

**Fig. 2:**
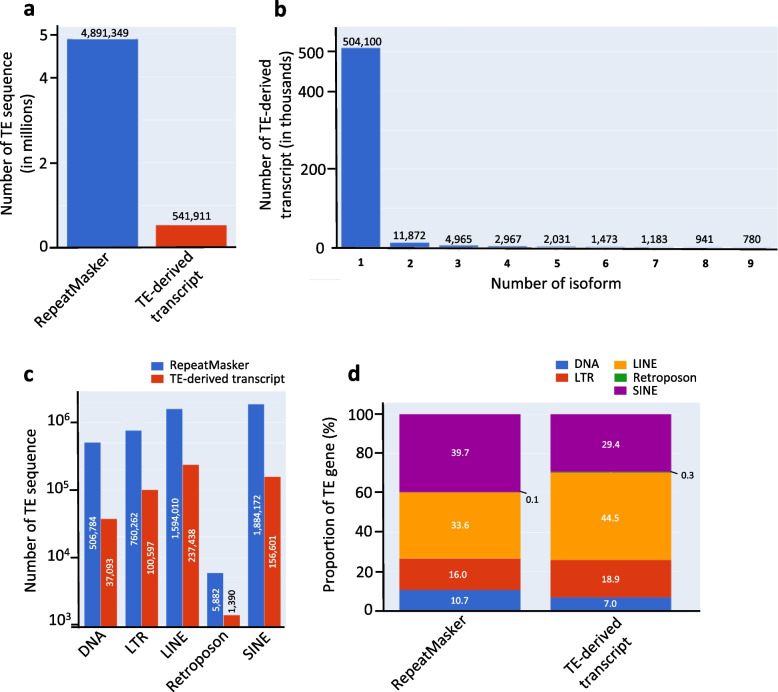
Summary of the TE-derived transcript annotation. **a** The number of TEs and TE-derived transcripts in the RepeatMasker and TE-derived transcript annotations. **b** Number of TE-derived isoforms corresponds to the number of TE-derived transcripts in the TE-derived transcript annotation. **c** Comparison of the number of TE sequences in each TE sumperfamily between RepeatMasker and TE-derived transcript annotations. **d** Comparison of the proportion of TE sequences in each TE superfamily between RepeatMasker and TE-derived transcript annotations

The total number of TE-derived transcripts in the TE annotation was 535,724. Of the TEs annotated using RepeatMasker (4,891,349 TEs), one-ninth (535,724) were included in our annotation (Fig. 2a) [24]. This result indicates that one out of every nine TE-derived sequences in RepeatMasker is expressed and is a putatively functional transcript. Approximately, 94% (504,100) of the TE-derived transcripts contained only one isoform, indicating that there were no other alternative transcripts (Fig. 2b). Some protein-coding sequence-containing TE superfamilies, such as long-interspersed nuclear element (LINE), ERV, DNA, and TE-nonTE gene fusion transcripts, can produce several isoforms, suggesting that they are the main source of isoforms. Additionally, the number and percentage of TE-derived transcripts for each superfamily can be seen in Fig. 2c and d. The five main TE superfamilies were extracted from the “repClass” columns in the RepeatMasker annotation. If a TE-derived transcript overlapped with multiple superfamilies, it was classified according to the superfamily with the greatest overlap. When the percentages of superfamilies in RepeatMasker and our TE-derived transcript annotation were compared, long terminal repeat retrotransposons (LTR) and LINEs showed higher proportions of TE-derived transcripts in our annotation (19% and 44%, respectively) than those observed in the RepeatMasker annotation (15% and 32%, respectively) (Fig. 2d). In contrast, short interspersed nuclear elements (SINE), DNA transposons (DNA), and SVA retroposons (Retroposons) showed lower proportions of TE-derived transcripts in the TE-derived transcript annotation (29%, 7%, and 0.3%, respectively) than in the RepeatMasker annotation (39%, 10%, and 0.1%, respectively). Taken together, these data indicate that our TE-derived transcript annotation provides basic information about locus-specific, transcriptionally active TE-derived transcripts in humans.

The gene splicing process is very complex. The spliceosome, a ribonucleoprotein complex that catalyzes RNA splicing, recognizes specific sequences known as splicing acceptor sites (AG) and splicing donor sites (GT) [36]. To validate whether the isoforms in the TE-derived transcript annotation were accurately generated, we analyzed the gene annotation and TE-derived transcript annotation for these splicing sequence motifs. In the GENCODE reference nonTE gene annotation, the splicing acceptor and donor sites were highly conserved and matched the splicing consensus sequence (see Supplementary Fig. 3a). Next, we performed the same motif analysis in the TE-derived transcript annotation (Supplementary Fig. 3b). We found that splicing consensus sequences, as well as sequences that are preferentially located nearby splicing consensus sequences, were clearly present in the TE-derived transcript annotation. To rule out the possibility that the splicing consensus sequences of transcripts in the TE-derived transcript annotation came from reads of nonTE genes, we carried out the same motif analysis in the TE-derived transcript annotation without transcripts overlapping with nonTE genes (Supplementary Fig. 3c). These results were nearly identical to those of the two previous analyses, indicating that the isoforms of the TE-derived transcripts are reliable. These analyses confirmed that the splicing motifs in TE-derived transcript isoforms were consistent and reliable, regardless of their overlap with nonTE genes.

### The TE-derived transcript annotation provides high-resolution information about transcriptionally active TEs

**Fig. 3:**
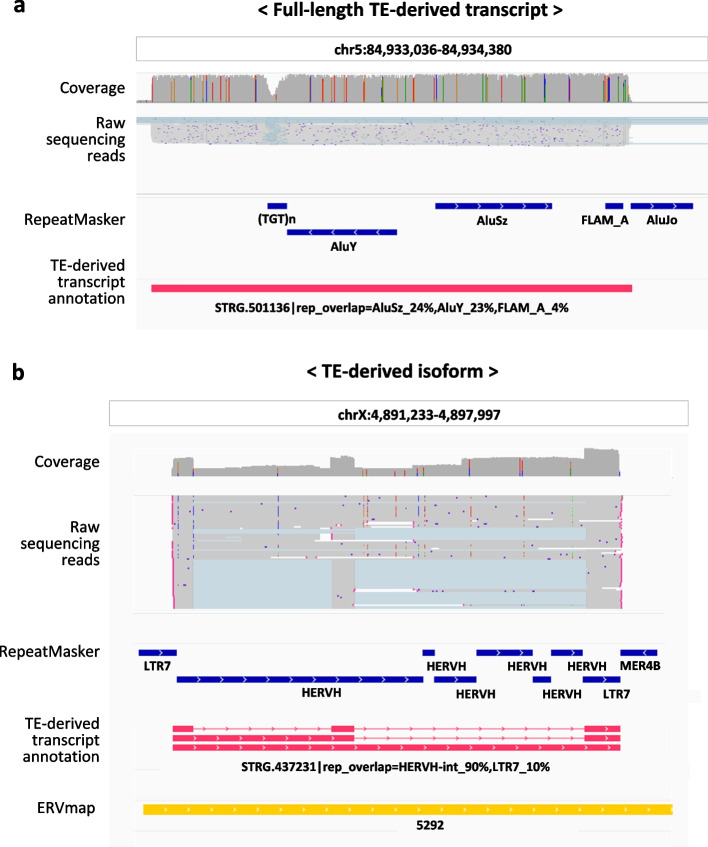
Genome browser view of novel TE-derived transcripts that are independent of nonTE genes. **a** Genome browser image of expressed reads overlapping with TE-derived transcript STRG.501136, along with copies of the TEs AluSz and FLAM_A. **b** Genome browser image of the expressed reads overlapping TE-derived transcript *STRG.437231* and its isoforms, combined with several copies of the TEs *HERVH* and *LTR7*. In **a** and **b**, the GENCODE nonTE gene annotations are shown in green, RepeatMasker annotations are shown in blue, TE-derived transcript annotations are shown in red, and ERVmap annotations are shown in yellow. In the annotation, a transcript with a line indicates an individual isoform in the same genome region, and a transcript with no line refers to the main transcript model in the same genome region. In the reads, sequences that match nucleotide sequences in the reference genome are colored gray, and sequences that do not match nucleotide sequences in the reference genome due to splicing are colored blue

**Fig. 4:**
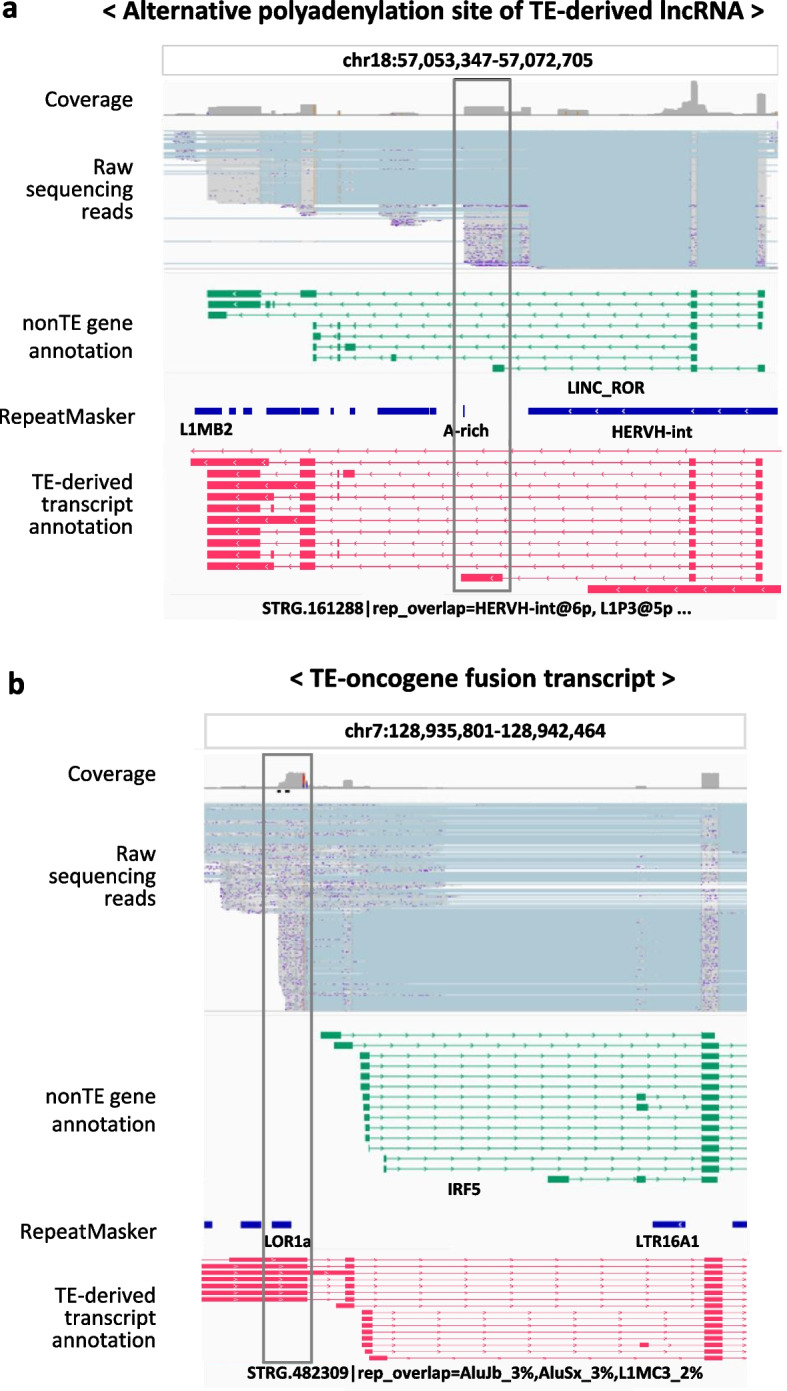
Genome browser view of TE-nonTE gene fusion transcripts and improved transcription end site (TES) annotation. **a** Genome browser image of the expressed reads overlapping with TE-derived transcript *STRG.161288*, which has a different polyadenylation site from the TE-derived lncRNA *LINC-ROR*. **b** Genome browser image of the expressed reads overlapping with TE-derived transcript *STRG.482309*, which includes copies of the *LOR1a* TE and the *IRF5* oncogene. The 17 th isoform of *STRG.482309* is relatively short and was not included in the genome browser view. In **a** and **b**, the GENCODE nonTE gene annotations are shown in green, RepeatMasker annotations are shown in blue, and TE-derived transcript annotations are shown in red. In the annotation, a transcript with a line indicates an individual isoform in the same genome region. In the reads, sequences that match nucleotide sequences in the reference genome are colored gray, and sequences that do not match nucleotide sequences in the reference genome due to splicing are colored blue

We identified four important characteristics that demonstrate the usefulness of our LR RNA-seq data-based TE-derived transcript annotation. First, we observed previously unannotated full-length TE-derived transcripts in the TE-derived transcript annotation (Fig. 3a and Supplementary Fig. 4a). The TE-derived transcript shown in Fig. 3a was 1270 base pairs (bp) long and contained three SINE subfamilies (AluY, AluSz, and FLAM-A). Second, we observed different isoforms of TE-derived transcripts in the TE-derived transcript annotation (Fig. 3b and Supplementary Fig. 4b). To further support the presence of multiple isoforms, isoform-specific expression levels and their relative abundances were visualized for the representative TE-derived transcripts in Supplementary Fig. 5a and b. As shown in Fig. 3b, a TE-derived transcript belonging to the ERV superfamily had three full-length isoforms in the TE-derived transcript annotation. ERVs, originating from retroviral proviruses, encode proteins essential for viral replication and survival and likely enable TE-derived transcripts containing ERV sequences to generate multiple isoforms [37]. These isoform-containing TE-derived transcripts were also matched to the region specified in the ERVmap database, suggesting that the expected regions contain intact, near-full-length proviral ERV [38]. In contrast, the RepeatMasker annotation identified this region as comprising nine separate repeat sequences: two LRT7 elements, six HERVH elements, and one MER4B element. Together, these data demonstrate that the TE-derived transcript annotation contains transcriptionally active TE-derived transcripts and their isoforms at unique loci. Thirdly, our analysis generated a more accurate mapping of the transcription end sites (TES) of TE-containing transcripts compared to the GENCODE nonTE gene annotation (Fig. 4a). The annotated TES for an isoform of the lncRNA transcript *LINC-ROR*, previously known as onco-lncRNA, is located at 57,063,555 bp on chromosome 18 in the nonTE gene annotation, but it is located at 57,062,614 bp on chromosome 18 in the TE-derived transcript annotation [39]. The shifted TES of the *LINC-ROR* isoform exactly matched some of the sequencing reads in the collected LR RNA-seq data. This result indicated that the assembled TE annotation successfully captured the TE-derived transcripts identified in the LR RNA-sequencing data. Finally, we confirmed that the known TE-nonTE gene fusion transcripts corresponded to previously reported oncogenic fusion events involving *LOR1a-IRF5* (Fig. 4b) [40, 41]. Notably, these fusions were not annotated in the nonTE gene annotation, highlighting the potential of TE-derived transcript annotation for uncovering novel transcript structures. Collectively, our study highlights the value of our long-read RNA-based TE-derived transcript annotation for refining known TE-containing transcripts and identifying novel isoforms and unannotated oncogenic fusion transcripts.

### Identification of cell type-specific, active TE transcripts

Next, we applied our TE annotation to scRNA-seq data to identify tissue and cell type-specific expression of TE transcripts in various human cells. For this analysis, Genotype-Tissue Expression (GTEx) scRNA-seq data were utilized, which included 3 or 4 samples from 8 human tissue types (breast, esophagus mucosa, esophagus muscularis, heart, lung, prostate, skeletal muscle, and skin) collected from 16 healthy participants [32].

**Fig. 5:**
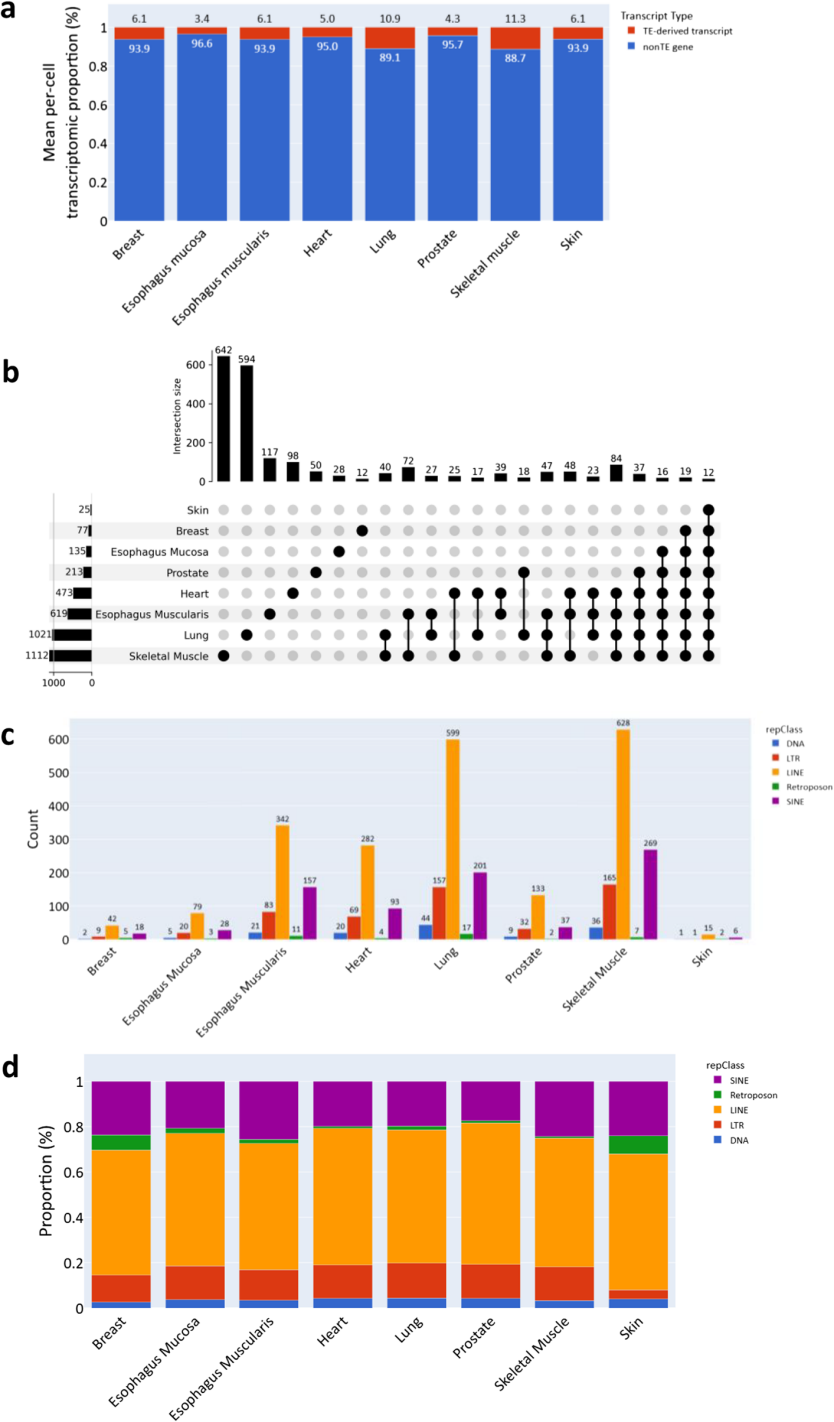
Tissue-level analysis of TE-derived transcript distribution in single-cell RNA-sequencing data. **a** Average proportion of TE-derived transcripts in scRNA-seq data by tissue. Each bar represents the transcriptomic composition of a given tissue in terms of TE-derived transcripts and non-TE genes. The proportion of TE-derived transcripts relative to the total number of expressed features was computed for each cell and averaged by tissue. **b** UpSet plot illustrating the shared and tissue-specific TE-derived transcripts identified from scRNA-seq data. Only transcripts with a total normalized expression (calculated using the sc.pp.log1p function from the Scanpy package) ≥ 100 within each tissue were considered. Each dot set represents a combination of tissues that share a set of TE-derived transcripts. The intersection size refers to the number of TE-derived transcripts shared among the indicated tissues. Intersections involving fewer than 10 transcripts were excluded from the visualization. **c** Bar plot showing the number of TE-derived transcripts per tissue, grouped by TE class. TE-derived transcripts were selected based on the same criteria as in **b** (normalized expression ≥ 100 per tissue). If multiple TE classes overlapped with a single transcript, the class with the largest overlap was assigned. **d** Stacked bar plot representing the relative proportions of TE classes among tissue-level TE-derived transcripts shown in **b** and **c**

Prior to the cell type-level analysis, we first investigated the tissue-level transcriptomic composition. For each cell, the proportion of TE-derived transcripts relative to the total number of expressed transcripts was calculated and subsequently averaged within each tissue (Fig. 5a). Among the examined tissues, skeletal muscle and lung exhibited the highest proportions of TE-derived transcripts, with over 10% of the transcriptome originating from TEs. On average, TE-derived transcripts accounted for 6.7% of the total transcriptome (TE + nonTE) across all tissues. Despite inter-tissue variability, the proportions were relatively consistent. To further evaluate transcript-level similarities and differences among tissues, we identified TE-derived transcripts with a total normalized expression ≥ 100 in each tissue (see Fig. 5b). Consistent with the overall abundance, skeletal muscle and lung harbored the largest numbers of unique transcripts, while 12 TE-derived transcripts were found to be shared across all 8 tissues. Examination of the number of unique molecular identifiers (UMIs) per cell across tissues revealed that the average sequencing depths in skeletal muscle and lung were lower than those in prostate, heart, and esophagus mucosa (Supplementary Fig. 6a). This result suggests that the number of TE-derived transcripts detected in each tissue is not linearly correlated with sequencing depth. Additionally, we investigated the number and proportion of TE-derived transcripts for each TE superfamily across tissues (Fig. 5c and d). As in Fig. 2c and d, the five major TE superfamilies were defined based on the “repClass” column from the RepeatMasker annotation, with each TE-derived transcript assigned to the superfamily exhibiting the greatest degree of overlap in cases of multiple overlaps. While the relative proportions of each superfamily varied slightly across tissues, the overall patterns were consistent. Notably, LINE elements dominated the TE-derived transcriptome in all tissues, with an average proportion of 58.5%, which is substantially higher than their representation of 44.5% in the full set of annotated TE-derived transcripts (Figs. 2d and 5d).

**Fig. 6:**
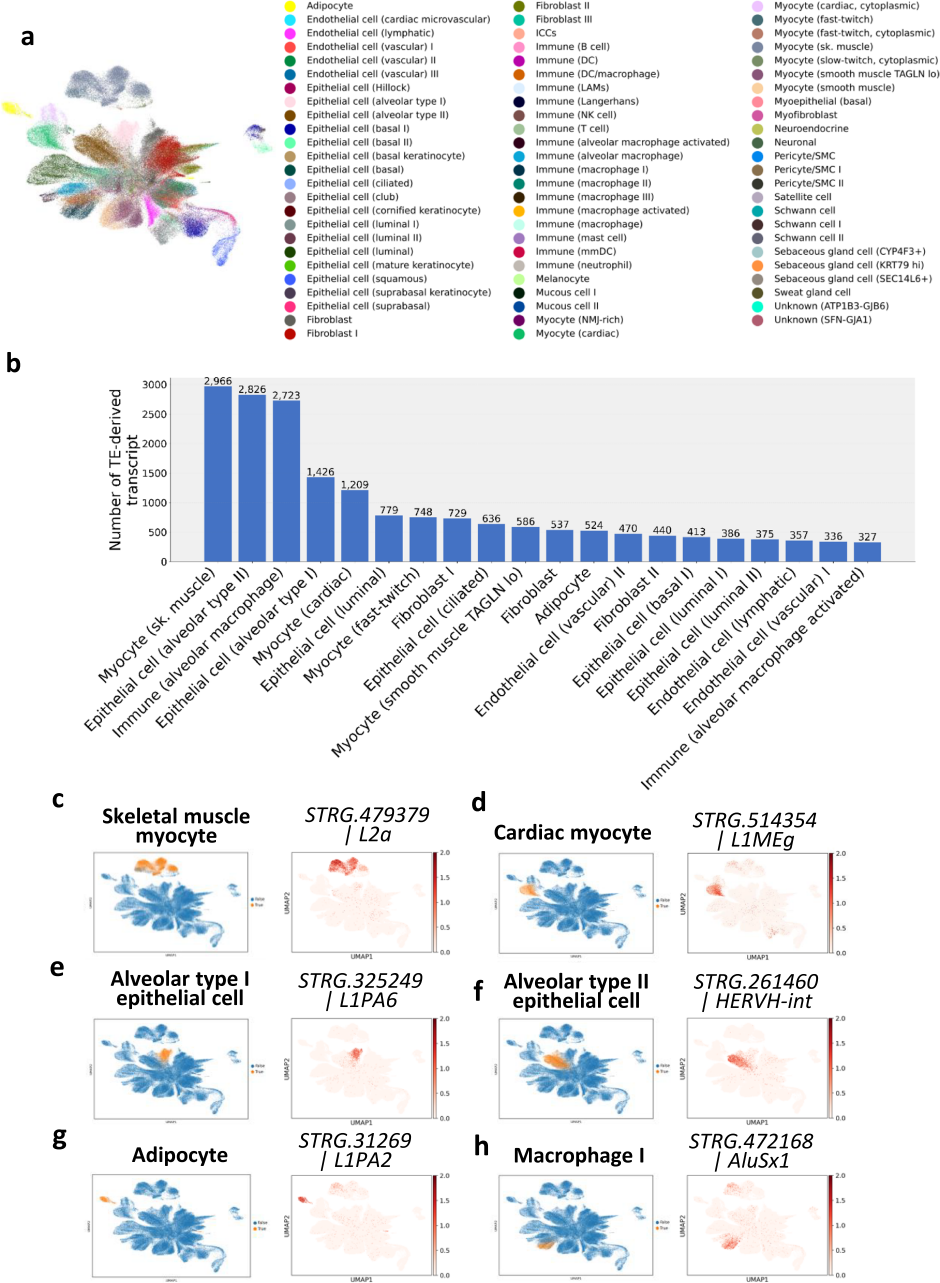
Cell type-specific landscape of TE-derived transcripts in single-cell RNA-sequencing data. **a** A UMAP plot of the total cells in the Genotype-Tissue Expression (GTEx) dataset clustered by both nonTE- and TE-derived transcripts. Cells were clustered into 74 types, which were annotated in previous studies. Each color represents a cell type. **b** Top 20 cell types ranked by the number of differentially expressed TE-derived transcripts. **c**, **d**, **e**, **f**, **g**, **h** Expression patterns of the cell type-specific TE-derived transcripts in a selected set of cell types. The left plot indicates the location of a specific cell type on a UMAP embedding, colored orange. Plots on the right side shows the expression levels of cell-type-specific TE-derived transcripts for **c** skeletal muscle myocytes, **d** alveolar type II epithelial cells, **e** cardiac myocytes, **f** adipocytes, **g** alveolar macrophage cells, and **h** T cells

After the tissue-level analysis, we next performed a cell type-level investigation. A total of 74 clusters were identified using both nonTE genes and TE-derived transcripts and were annotated based on previously reported cell type information from the GTEx study. These clusters were largely consistent with the established annotations (Fig. 6a). Differential expression analysis revealed a total of 16,459 TE-derived transcripts across all cell types (Supplementary Data S2, Supplementary Data S3). Among the 74 cell types, skeletal muscle myocytes exhibited the highest number of differentially expressed TE-derived transcripts, followed by alveolar type II epithelial cells and alveolar macrophages (Fig. 6b). Similar to the tissue-level analysis, the number of detected TE-derived transcripts across cell types and the sequencing depth per cell appeared to be uncoupled (Supplementary Fig. 6b), suggesting that TE detection is not solely dependent on sequencing depth. Several of these TE-derived transcripts exhibited distinct cell type-specific expression patterns (Fig. 6c, d, e, f, g, h), similar to those observed in nonTE genes. These findings highlight the biological relevance of TE-derived transcripts and suggest their potential role in defining cell identity.

To examine broader transcriptional trends, the 74 cell types were further grouped into 5 major categories: myocyte, epithelial, endothelial, immune, and others. Among these, the myocyte and epithelial groups displayed a higher mean number of TE-derived transcripts compared to the immune and other groups (Supplementary Fig. 6d). Interestingly, at this major group level, the distributions of sequencing depth and TE-derived transcript abundance appeared to covary (Supplementary Fig. 6c). Collectively, these results demonstrate that TE-derived transcripts exhibit distinct and biologically meaningful expression patterns across tissues, cell types, and broader cell-type groups.

## Discussion

TEs constitute a significant portion of the human genome, and their transcripts have been shown to play key roles in various biological and evolutionary processes and in diseases, such as cancer. Despite their importance, current TE annotations, such as RepeatMasker, are limited in their ability to precisely quantify TE-derived transcripts because they lack detailed exon- and transcript-level information. In addition, current tools for quantifying TEs at the single-cell level cannot measure transcriptionally active TEs at unique loci across diverse cell types. To overcome this limitation, we aimed to identify cell type-specific, transcriptionally active TE-derived transcripts. Using LR RNA-seq data from 2596 publicly available samples, we generated a comprehensive TE-derived transcript annotation encompassing 535,724 transcripts, including isoforms generated through alternative splicing. The robustness of this approach was demonstrated by identifying known TE-nonTE gene fusion transcripts and established genes. Using this refined annotation, we conducted a scRNA-seq analysis that revealed distinct TE-derived transcript expression patterns across various cell types, thereby highlighting the cell type-specific activity of the TEs and their potential biological significance.

The TE-derived transcript annotation constructed in this study revealed several core features that could only be revealed by using a long-read RNA-based comprehensive transcriptome assembly. We identified previously unknown full-length TE-derived transcripts consisting of one or more copies of TEs. In addition, we observed not only TE-derived transcripts but also their isoforms. ERVs and LINEs, two representative superfamilies of TEs, are known to have multiple protein-coding sequences capable of producing more than one protein, and the alternative splicing of transcripts from these TE classes is well documented [42]. Considering that some TE-derived proteins contribute to fetal morphogenesis and cancer progression [4, 6], characterization of the isoforms from TE-derived transcripts at the locus-specific level is essential for investigating their functional effects on biological processes and diseases. Collectively, these novel, transcriptionally active TE-derived transcripts improve the accuracy of quantification of TE-derived transcripts in transcriptome data analysis.

Furthermore, our annotation confirmed previously annotated TE-containing transcripts, such as TE-containing long non-coding RNAs and TE-nonTE gene fusion transcripts. Interestingly, we observed that one of the isoforms of the lncRNA transcript *LINC-ROR* in the TE-derived transcript annotation had a TES that resulted in a longer transcript than in the nonTE annotation. The TES in the TE-derived annotation position precisely matched the end site of overlapping reads from 2596 LR RNA-seq data, further validating the reliability of the TE-derived annotation, which leverages the advantage of long-read data. In addition, the TE-derived transcript annotation allowed us to reconfirm a previously reported onco-expansion phenomenon in Hodgkin’s lymphoma: the *LOR1a-IRF5* fusion transcript [40]. Interestingly, we confirmed that this TE-oncogene fusion transcript was highly enriched in B-lymphoblastoid cell lines. Given the established role of IRF5 in early B-cell activation and differentiation, the *LOR1a-IRF5* fusion transcript may contribute to lymphocyte proliferation, potentially by mimicking or dysregulating IRF5 function in both normal and cancerous cells [43]. With the validation of the TE-oncogene fusion transcript as a proof of concept, comprehensive identification of potential TE-gene fusion transcripts in the TE-derived transcript annotation holds promise for advancing research on a wide range of diseases.

One of the key findings of this study was the identification of cell type-specific TE-derived transcripts at the individual transcript level. Although previous studies have shown that TE-derived transcripts are expressed heterogeneously across various cell types at the TE family level, our TE annotation enabled the identification of cell type-specific TE-derived transcripts at precise genomic loci, which was supported by experimentally validated expression, thus offering a clearer view of transcriptionally active TEs [15, 16]. In particular, a significant number of TE-derived transcripts were highly expressed in myocytes among the 74 cell types analyzed. Several studies have shown that TE-derived transcripts correlate with muscle disease and development; however, their exact biological functions remain unknown. The results of previous studies and ours indicate that the function of TE-derived transcripts in muscle needs further investigation [44, 45]. Exploring the presence and function of TE-derived transcripts in muscle cells and other cell types in greater depth could provide valuable insights into their broader biological significance. Considering these findings, TE-derived transcript annotation may provide interesting clues not only in various types of healthy cells but also across various disease conditions. In particular, the applicability of this TE-derived transcript annotation in cancer is promising because previous studies have shown that many TEs are highly expressed in cancer due to hypomethylation, which is a hallmark of cancer [6, 46]. Cell-to-cell fusion and onco-exaptation, resulting from TE-derived protein production, are typical outcomes of TE-derived transcript expression in cancer [40, 47]. Hence, a long-read-based TE-derived transcript annotation that provides isoform information and expressed transcript information may be helpful in elucidating the potential mechanisms by which TE-derived transcripts promote cancer progression.

One limitation of our current pipeline is the potential under-detection of short micro-exons (< 30 bp), which are known to be challenging to align accurately using default long-read alignment parameters [48]. While our conservative strategy was designed to maximize the specificity of TE-derived transcript detection, especially in repetitive regions, this may have come at the cost of reduced sensitivity for small exon structures. Future improvements may involve using more exon-sensitive aligners such as deSALT, adjusting alignment parameters (e.g., lower gap penalties), or incorporating known junction annotations to enhance exon recovery and transcriptome completeness [49].

## Conclusion

Our study focused on identifying cell type-specific transcriptionally active TE-derived transcripts at unique genomic loci using comprehensive LR RNA-seq datasets. We successfully validated splicing events and uncovered key features that emphasize the reliability of using long-read data for TE-derived transcript analysis. By constructing a detailed TE transcript annotation, we identified several TE-derived transcripts specific to individual cell types, thereby providing new insights into their functional relevance. Our findings significantly advance our understanding of TE-derived transcripts and provide researchers a valuable resource for further investigation.

## Supplementary Information

Additional file 1: Supplementary Fig. 1. Flow chart for TE selection criteria. This flowchart outlines the criteria used to classify TE-overlapping reads into four distinct categories based on their genomic location relative to nearby nonTE genes and TE genes. Intergenic (Category 1): Reads that overlap exclusively with intergenic TE regions and do not overlap with any nonTE genes. Intragenic (Category 2): Reads that overlap with both nonTE genes and intragenic TE regions, with at least 80% of the read aligned to the TE region. Spanning both TE and nonTE genes (Category 3): Reads where one part overlaps with a nonTE gene and another part overlaps with an intergenic TE region, indicating potential TE-nonTE gene fusion transcripts. The others (Category 4): Reads that do not fit into the previous three categories but still overlap with TE sequences. Supplementary Fig. 2. Quantitative profiling of transposable element-overlapping reads across genomic selection categories and tissue types. a. Proportion of TE-overlapping reads relative to the total number of uniquely mapped reads across four genomic selection categories in 2,596 LR RNA-seq samples. Each bar represents the percentage of reads overlapping transposable elements (TEs) under one of four classification strategies: intergenic, intragenic, spanning both TE and non-TE genes, and others. The “others” category includes all reads not assigned to the previous three categories. b. Tissue-specific distribution of genomic categories within the subset of TE-overlapping reads. Stacked bar plot showing the relative proportion of TE-overlapped reads assigned to each category across different tissue types. Category definitions are consistent with those described in panel a. The number of long-read RNA-seq samples used for each tissue was as follows: blood (*n = 36*), brain (*n = 50*), heart (*n=18*), liver (*n=7*), lung (*n=7*), oral (*n = 64*), ovary (*n = 9*), and stomach *(n = 8*). Supplementary Fig. 3. Splicing site validation for transcripts in the TE-derived gene annotation. a. Motif analysis of splice acceptor and donor sites in the nonTE gene annotation. The letters in each position indicate the nucleotides (A, T, C, and G), and the height of each letter represents the Shannon entropy (the relative frequency of nucleotides). b. The same as in a, but for the TE-derived transcript annotation. c. Similar to a, but here we used the TE-derived transcript annotation without the nonTE gene annotation. Supplementary Fig. 4. Verification of features of the TE-derived transcript annotation using Genome Browser view. a. Genome browser image of expressed reads overlapping TE-derived transcript *STRG.21148*, which was combined with copies of the TEs* LIMA3* and *AluSq2*. b. Genome browser image of the expressed reads overlapping TE-derived transcript STRG.319513 and its isoform, which was combined with copies of the TEs *HERV4_l* and*MER*. In a–d, the GENCODE nonTE gene annotations are shown in green, the RepeatMasker annotations are shown in blue, the TE-derived transcript annotations are shown in red, and ERVmap annotations are shown in yellow. In the annotation, a transcript with a line and an arrow refers to individual transcripts in the same genome region, and a transcript with no line refers to the main transcript model in the same genome region. In the reads, sequences that match nucleotide sequences in the reference genome are colored gray, and sequences that do not match nucleotide sequences in the reference genome due to splicing are colored blue. Supplementary Fig. 5. Isoform-level expression and relative abundance of TE-derived transcripts. a. Read counts for each isoform of selected TE-derived transcripts across four representative genes. b. Relative abundance for the same isoforms shown in a. The TE-derived transcripts, shown from left to right, correspond to those previously presented in Figure 3b, 4a, 4b, and Supplementary Figure 4b. Supplementary Fig. 6. Comparison of total unique molecular identifier (UMI) counts and TE-derived transcript abundance across tissues, cell types, and cell type groups. a-c. Boxplots showing the distribution of total UMI counts per cell across different biological groups. Each point represents a single cell, and UMI values were clipped at the 5th and 95th percentiles for visualization clarity. Cells were grouped by a) tissue, b) the top 20 cell types with the highest number of differentially expressed TE-derived transcripts, and **c)** five major cell type groups : myocytes, epithelial cells, endothelial cells, immune cells, and others. The “others” group comprises cell types that do not belong to the first four groups. d. Box plot showing the number of differentially expressed TE-derived transcripts in five major cell types. Each group included the number of cell types (n) indicated on the x-axis. The dashed red line represents the mean number of TE-derived transcripts in each group.

Additional file 2: Supplementary Data S1. TE-derived transcript annotation.

Additional file 3: Supplementary Data S2. List of TE-derived transcript specific to the 74 cell types in GTEx single-cell RNA-sequencing data. p-value, two-sided Student’s t-test.

Additional file 4: Supplementary Data S3. Genomic information about cell type-specific TE-derived transcripts in GTEx single-cell RNA-sequencing data.

## Data Availability

No datasets were generated or analysed during the current study.
