# Supplementary figures and images for: Identification of cell-type-specific, transcriptionally active transposable elements using long-read RNA-sequencing data-based comprehensive annotation
Source: Genomics Inform. 2025 Aug 6;23:17. doi: 10.1186/s44342-025-00048-1 (PMC12326599; doi:10.1186/s44342-025-00048-1)

Fig. S1

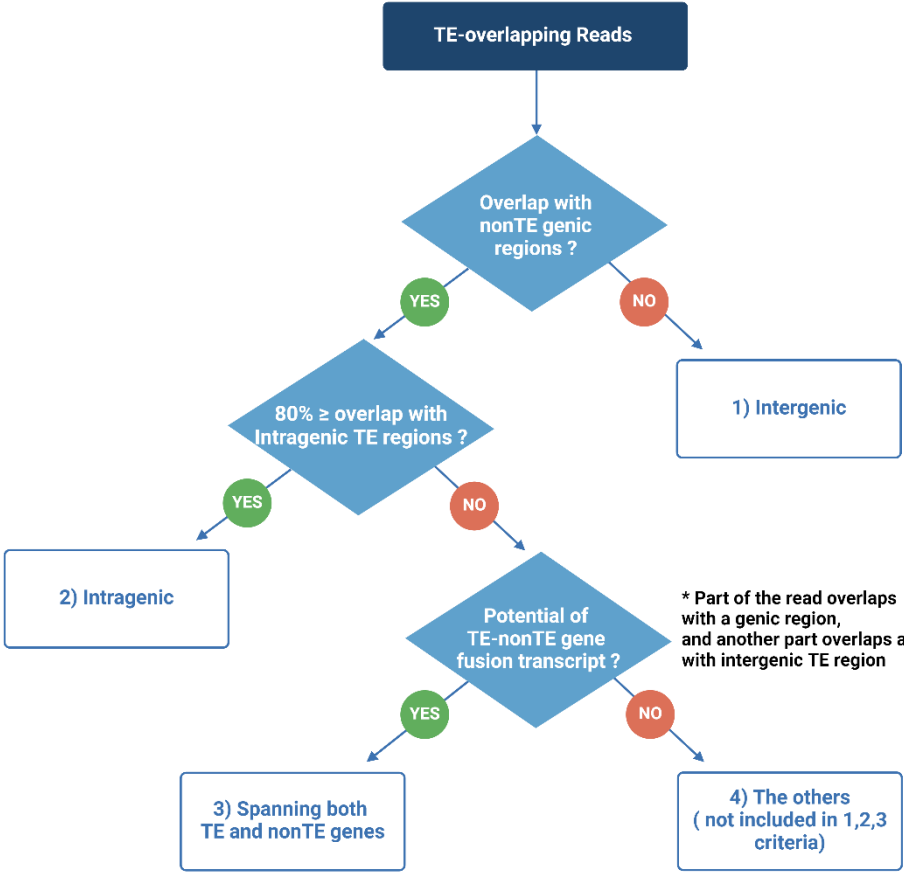

**Fig. S2**

**a**

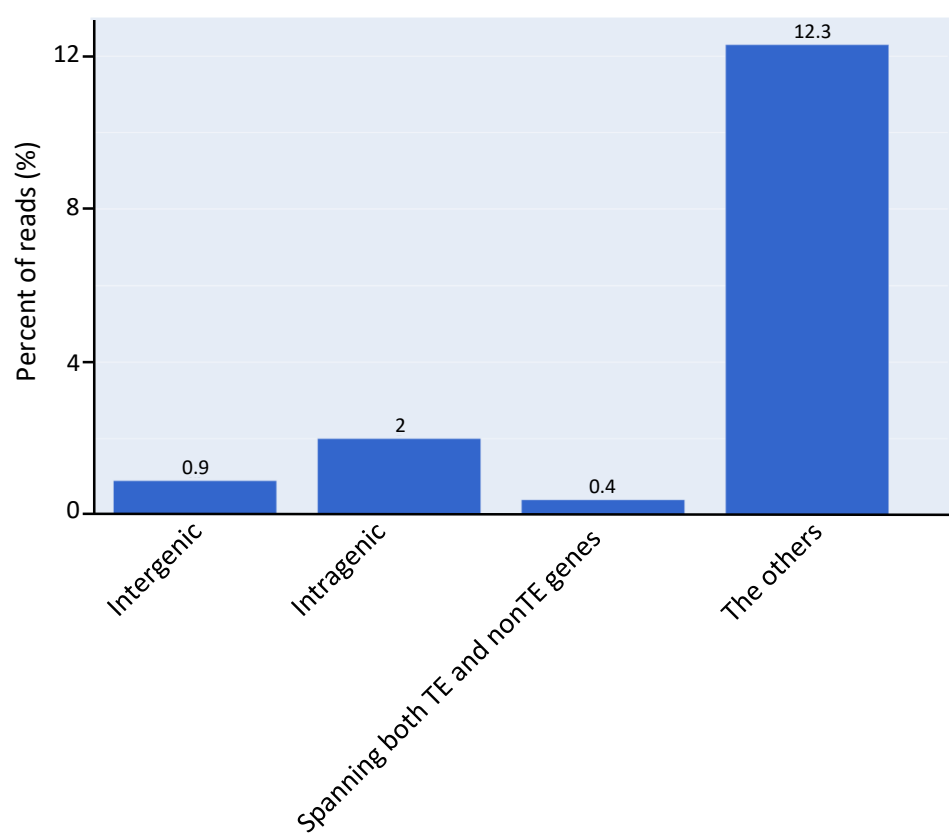

**b**

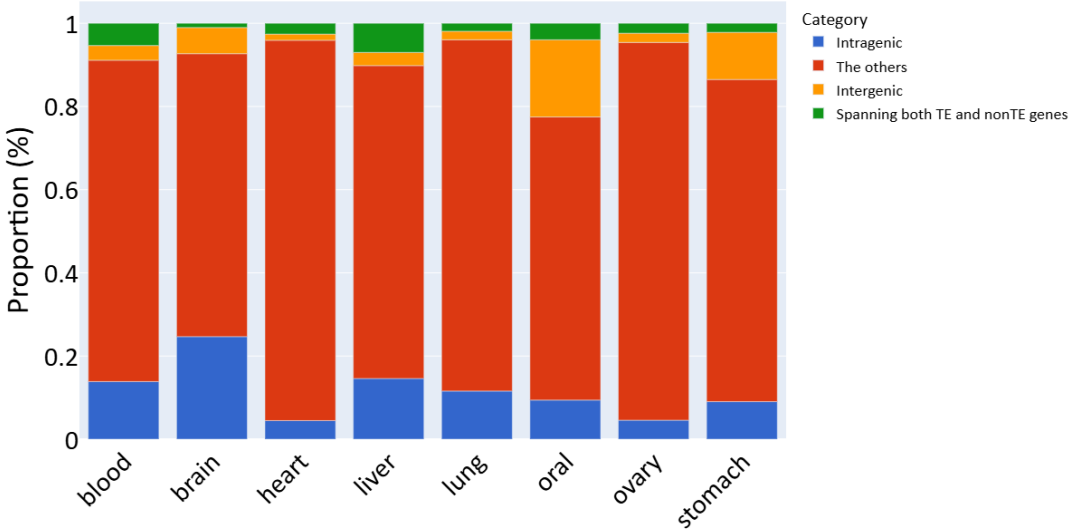

Fig. S3

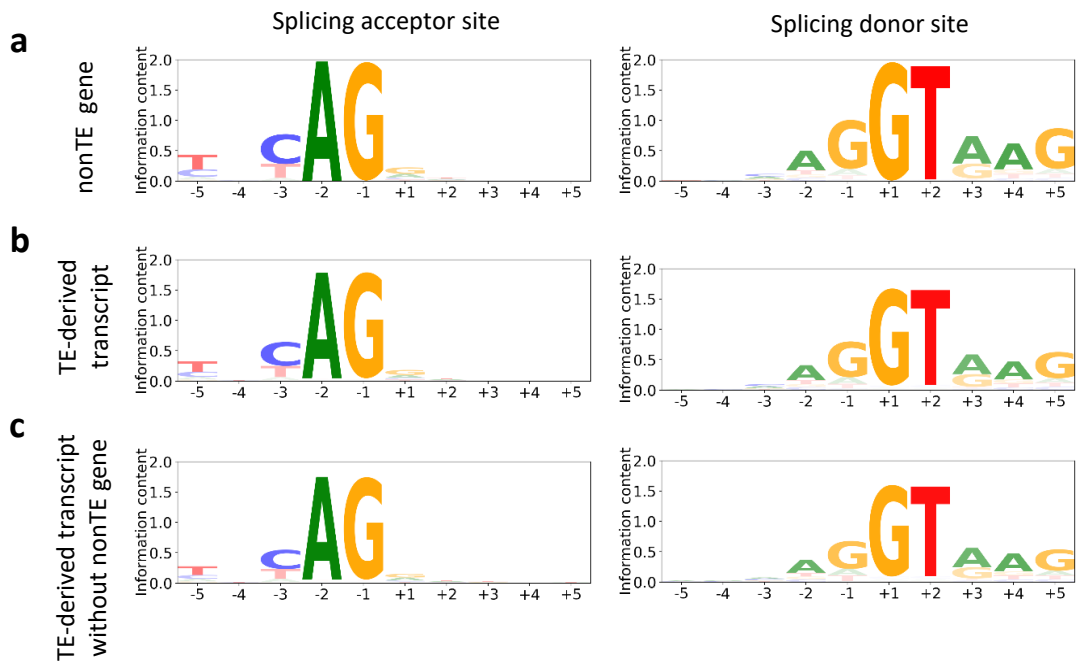

Fig. S4

a

< Full-length TE-derived transcript >

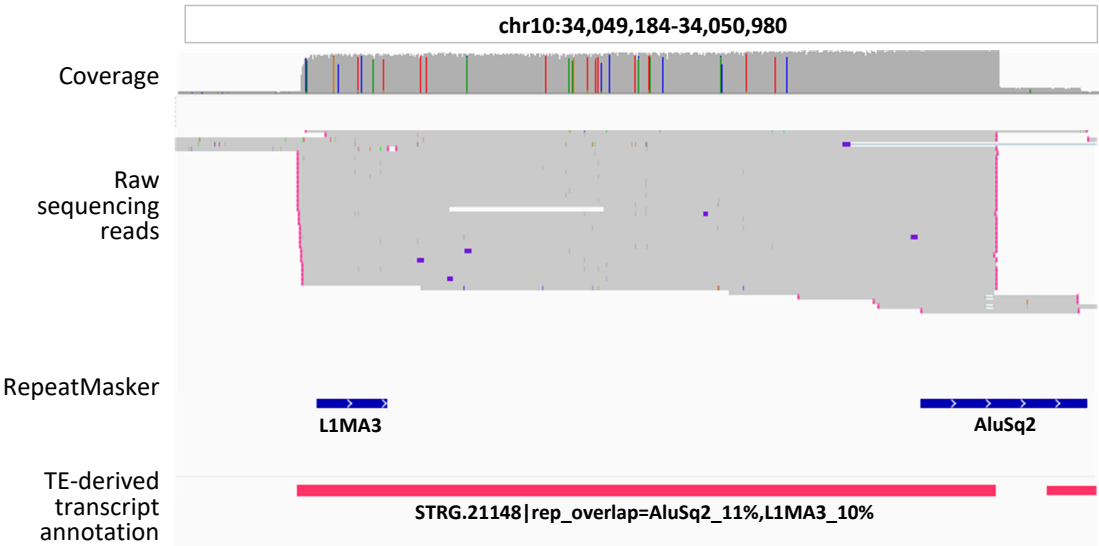

b

< TE-derived isoform >

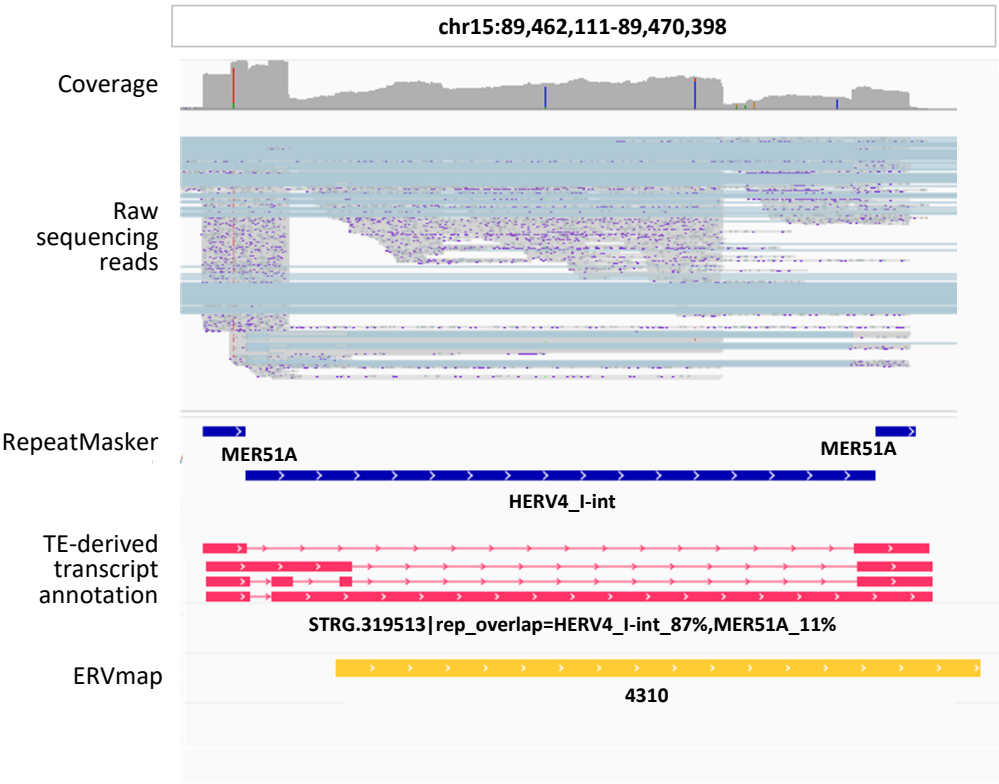

Fig. S5

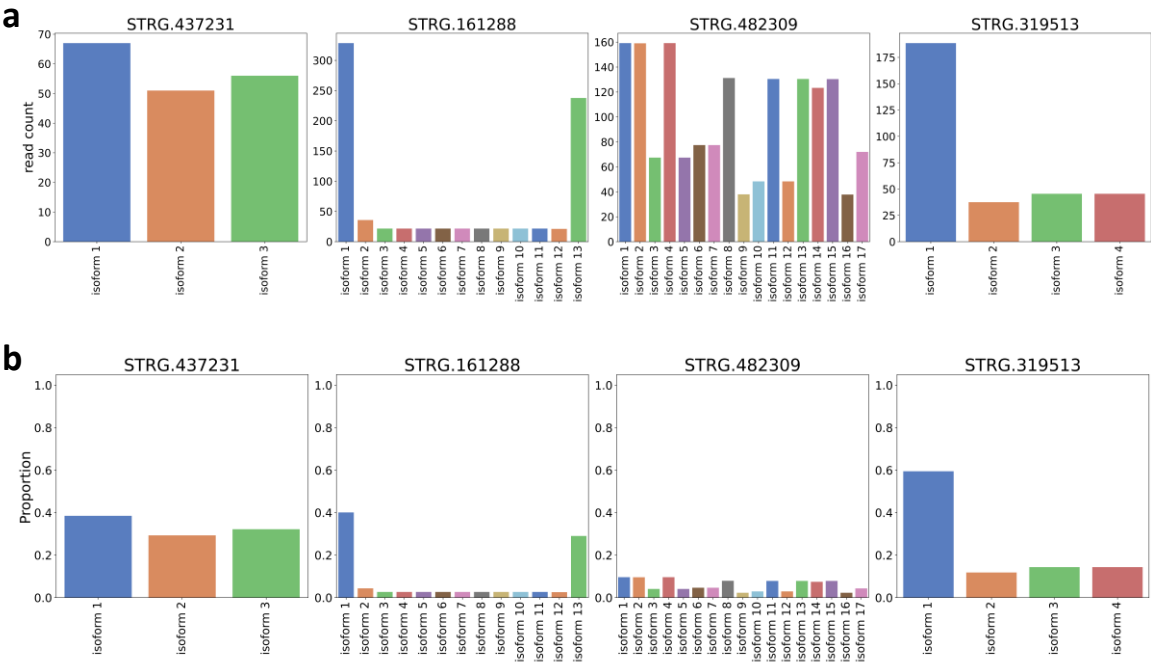

**Fig. S6**

**a**

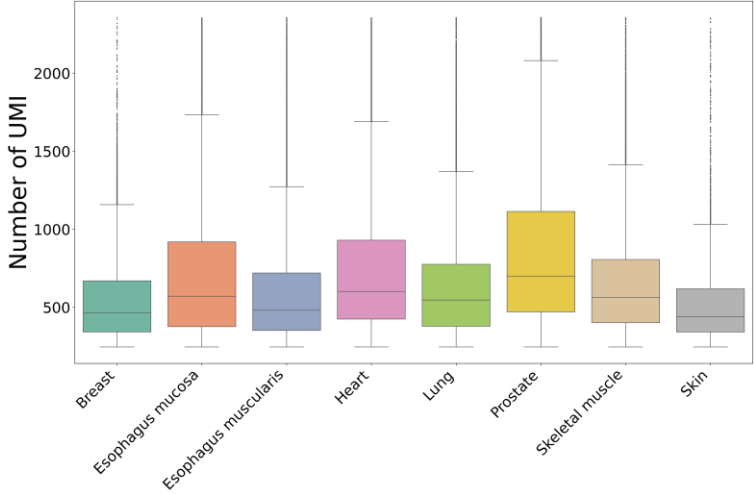

**b**

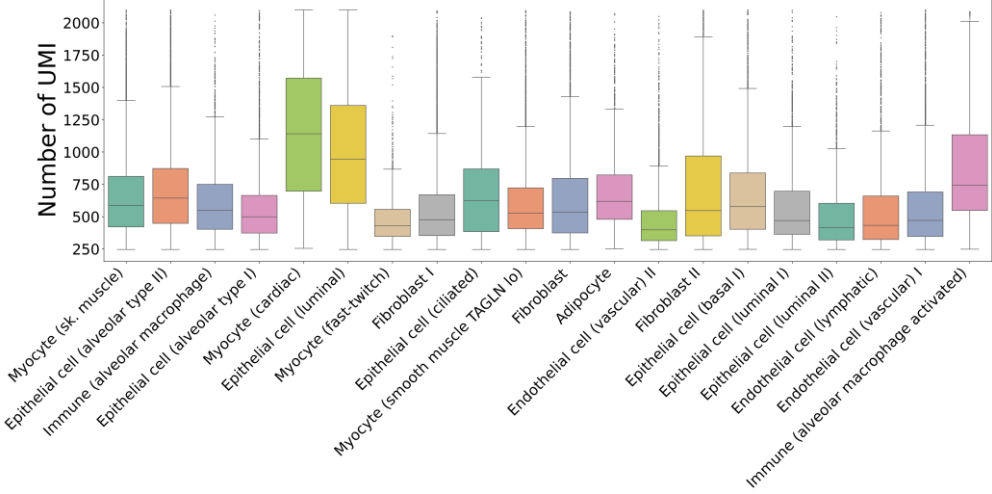

**c**

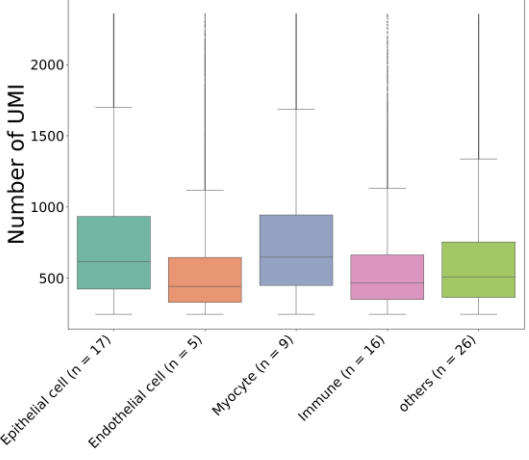

**d**

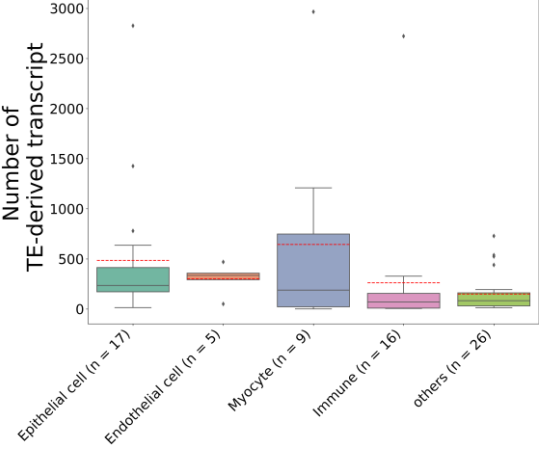

Supplement: Supplementary file 1 — Additional file 1: Supplementary Fig. 1. Flow chart for TE selection criteria. This flowchart outlines the criteria used to classify TE-overlapping reads into four distinct categories based on their genomic location relative to nearby nonTE genes and TE genes. Intergenic (Category 1): Reads that overlap exclusively with intergenic TE regions and do not overlap with any nonTE genes. Intragenic (Category 2): Reads that overlap with both nonTE genes and intragenic TE regions, with at least 80% of the read aligned to the TE region. Spanning both TE and nonTE genes (Category 3): Reads where one part overlaps with a nonTE gene and another part overlaps with an intergenic TE region, indicating potential TE-nonTE gene fusion transcripts. The others (Category 4): Reads that do not fit into the previous three categories but still overlap with TE sequences. Supplementary Fig. 2. Quantitative profiling of transposable element-overlapping reads across genomic selection categories and tissue types. a. Proportion of TE-overlapping reads relative to the total number of uniquely mapped reads across four genomic selection categories in 2,596 LR RNA-seq samples. Each bar represents the percentage of reads overlapping transposable elements (TEs) under one of four classification strategies: intergenic, intragenic, spanning both TE and non-TE genes, and others. The “others” category includes all reads not assigned to the previous three categories. b. Tissue-specific distribution of genomic categories within the subset of TE-overlapping reads. Stacked bar plot showing the relative proportion of TE-overlapped reads assigned to each category across different tissue types. Category definitions are consistent with those described in panel a. The number of long-read RNA-seq samples used for each tissue was as follows: blood (n = 36), brain (n = 50), heart (n=18), liver (n=7), lung (n=7), oral (n = 64), ovary (n = 9), and stomach (n = 8). Supplementary Fig. 3. Splicing site validation for trans [file 44342_2025_48_MOESM1_ESM.pdf]
